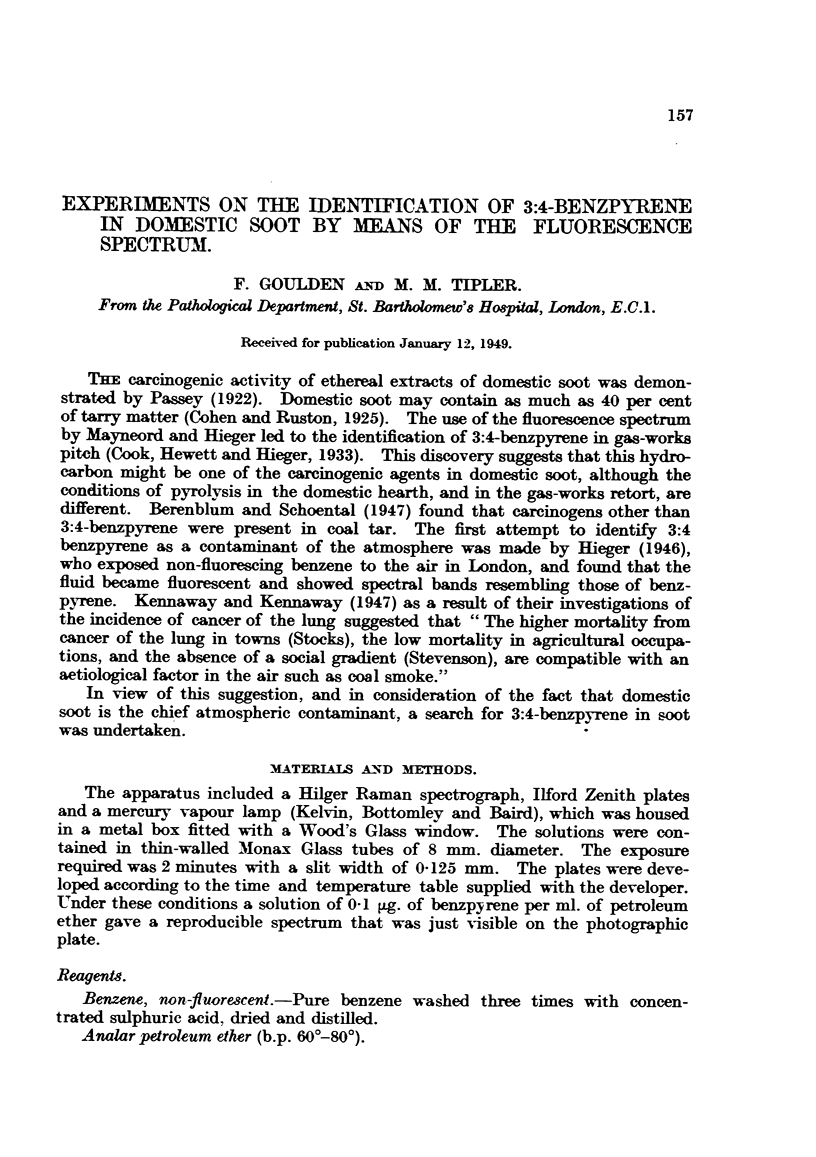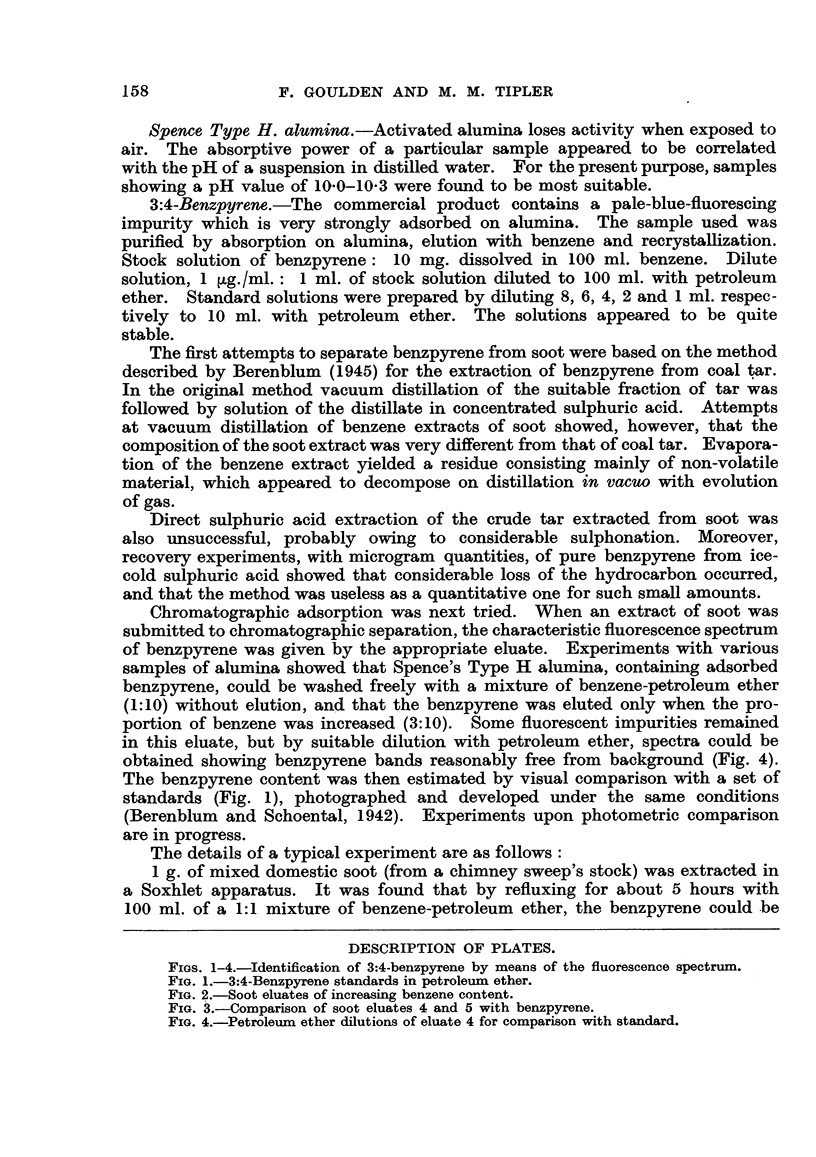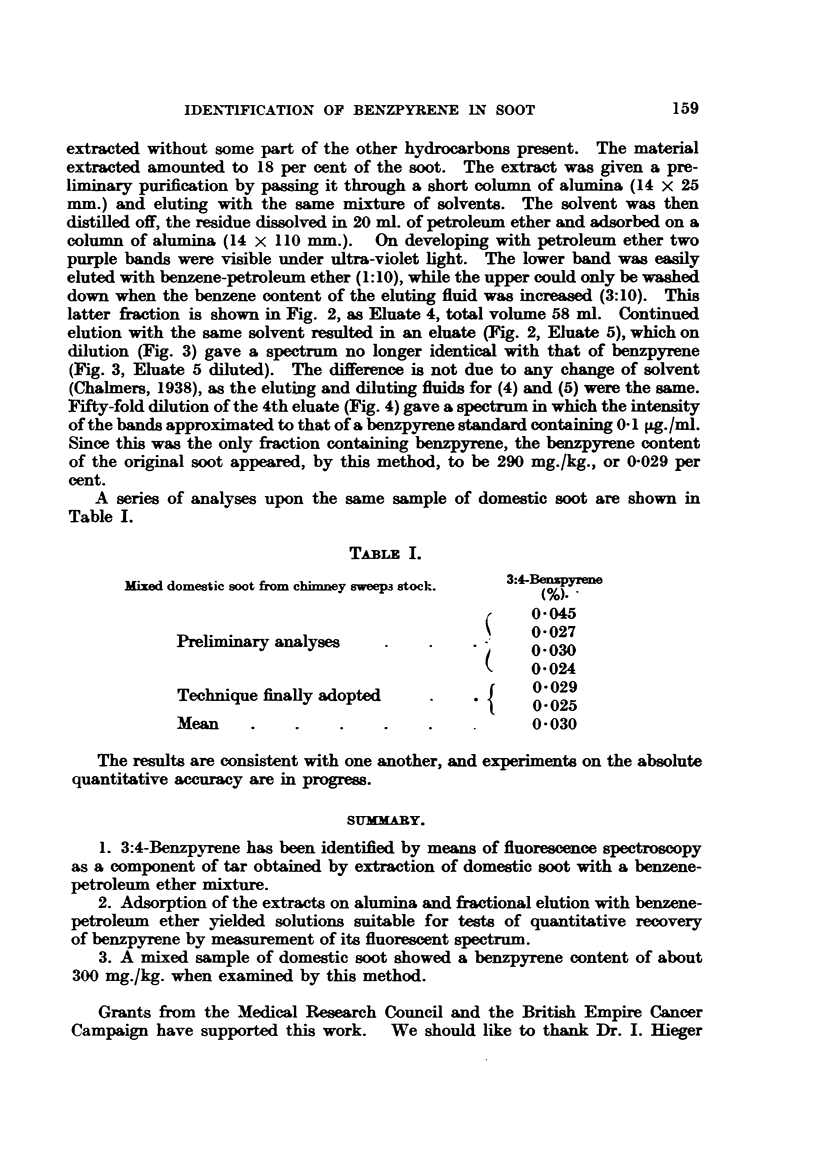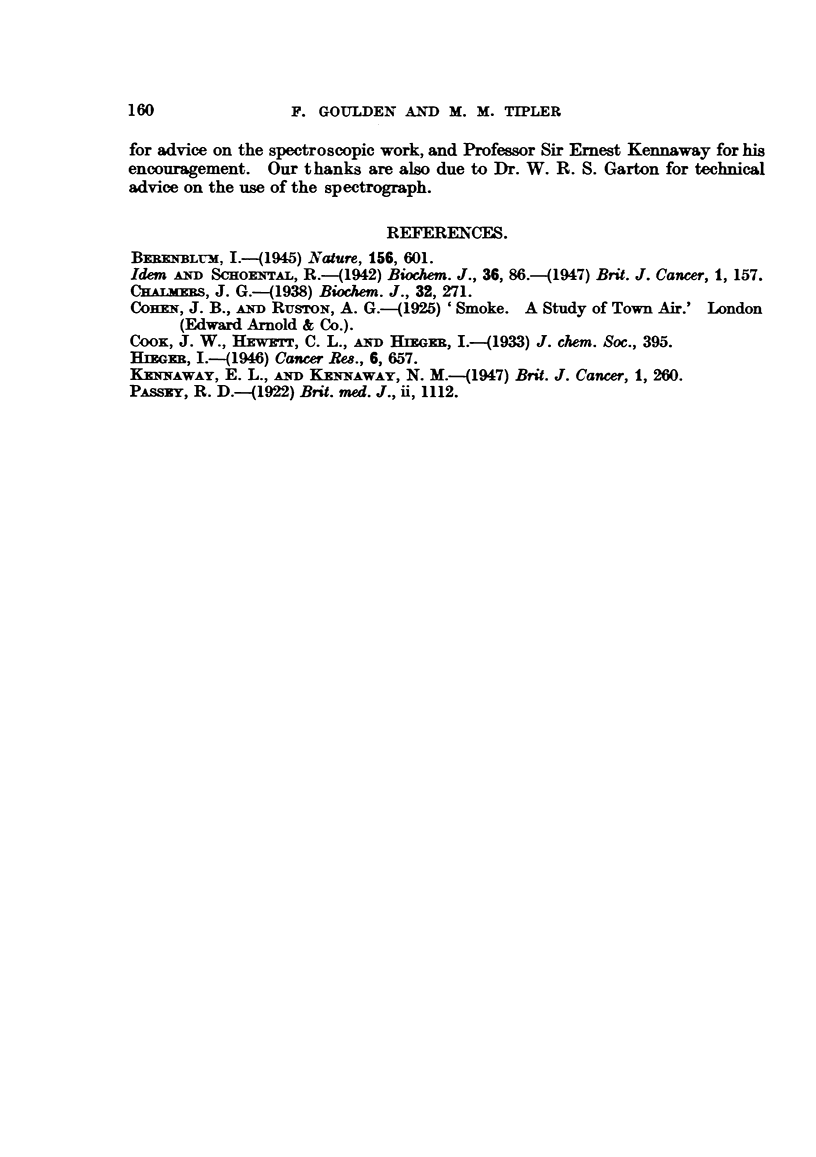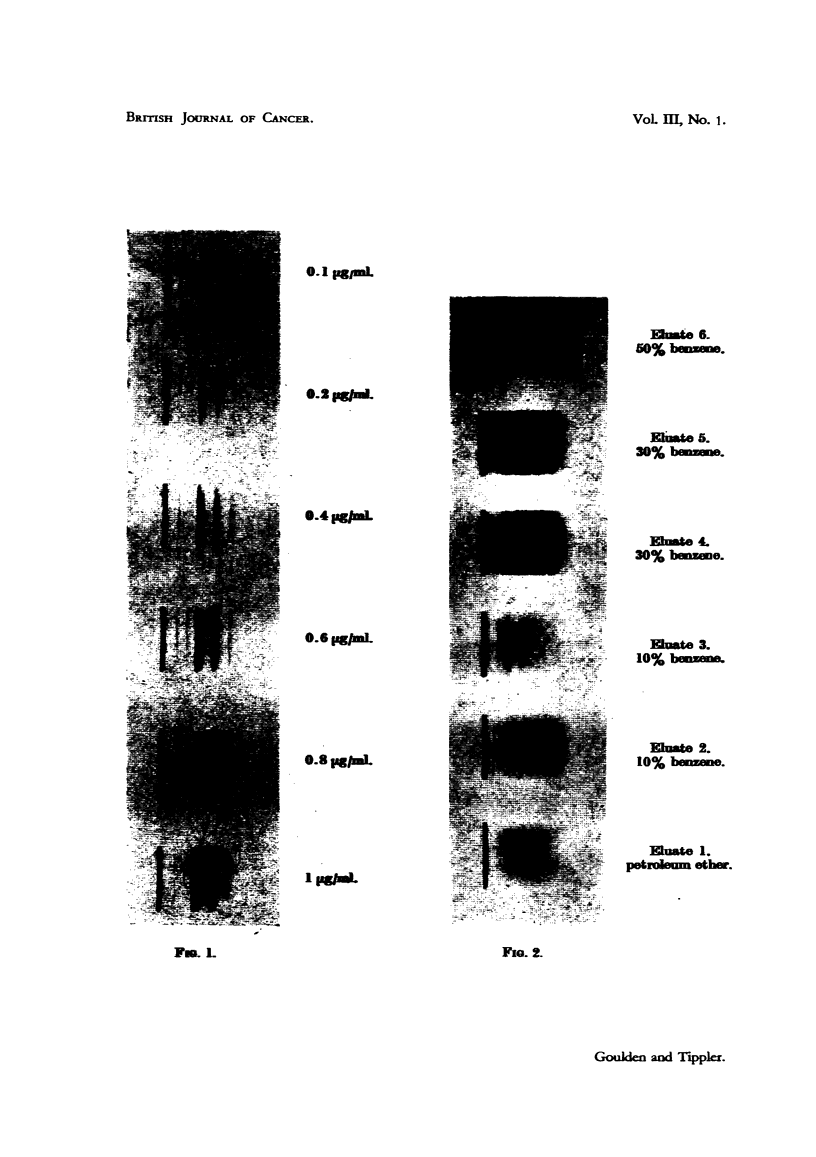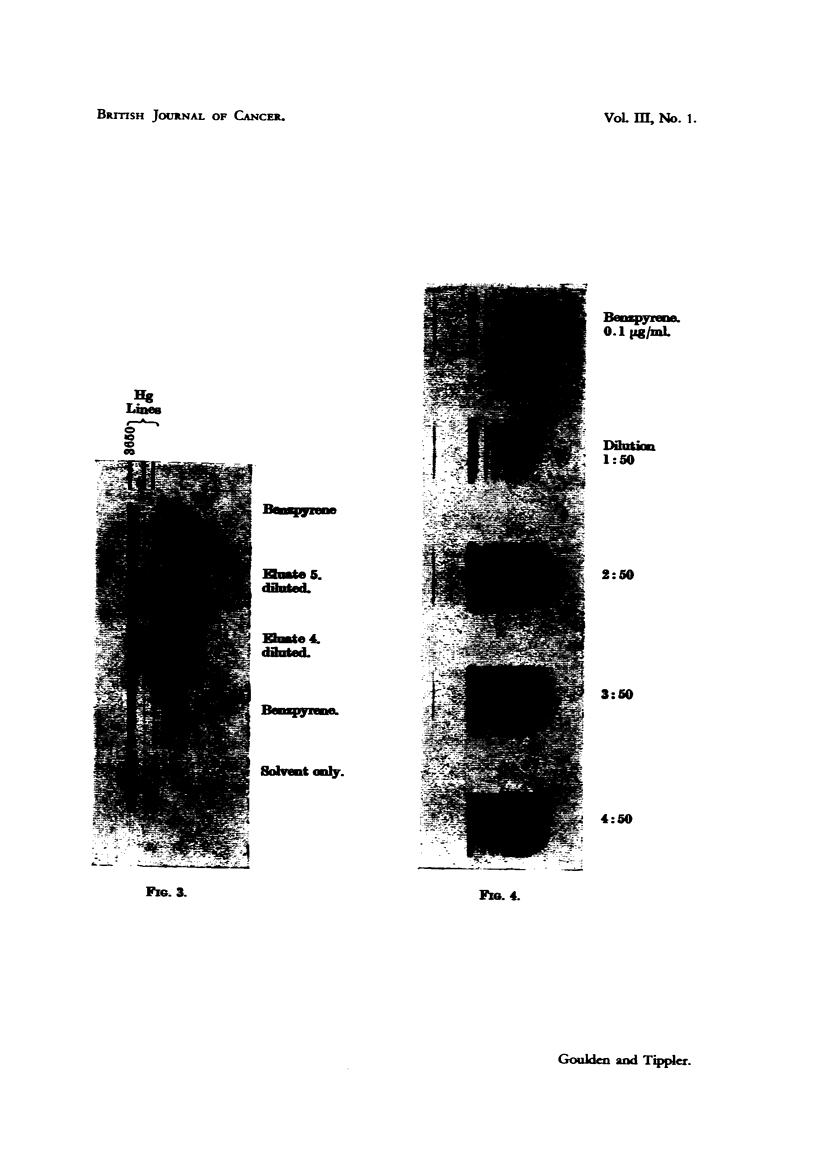# Experiments on the Identification of 3:4-Benzpyrene in Domestic Soot by Means of the Fluorescence Spectrum

**DOI:** 10.1038/bjc.1949.18

**Published:** 1949-03

**Authors:** F. Goulden, M. M. Tipler

## Abstract

**Images:**


### EXPERIMENTS ON THE IDENTIFICATION OF 3:4-BENZPYRENE IN DOMESTIC SOOT BY MEANS OF THE FLUORESCENCE SPECTRUM.

F. GOULDEN AND M. M. TIPLER.

*From the Pathological Department, St. Bartholomew's Hospital, London, E.C.*1.

Received for publication January 12, 1949.

THE carcinogenic activity of ethereal extracts of domestic soot was demonstrated by Passey (1922). Domestic soot may contain as much as 40 per cent of tarry matter (Cohen and Ruston, 1925). The use of the fluorescence spectrum by Mayneord and Hieger led to the identification of 3:4-benzpyrene in gas-works pitch (Cook, Hewett and Hieger, 1933). This discovery suggests that this hydrocarbon might be one of the carcinogenic agents in domestic soot, although the conditions of pyrolysis in the domestic hearth, and in the gas-works retort, are different. Berenblum and Schoental (1947) found that carcinogens other than 3:4-benzpyrene were present in coal tar. The first attempt to identify 3:4 benzpyrene as a contaminant of the atmosphere was made by Hieger (1946), who exposed non-fluorescing benzene to the air in London, and found that the fluid became fluorescent and showed spectral bands resembling those of benzpyrene. Kennaway and Kennaway (1947) as a result of their investigations of the incidence of cancer of the lung suggested that " The higher mortality from cancer of the lung in towns (Stocks), the low mortality in agricultural occupations, and the absence of a social gradient (Stevenson), are compatible with an aetiological factor in the air such as coal smoke."

In view of this suggestion, and in consideration of the fact that domestic soot is the chief atmospheric contaminant, a search for 3:4-benzpyrene in soot was undertaken.

### MATERIALS AND METHODS.

The apparatus included a Hilger Raman spectrograph, Ilford Zenith plates and a mercury vapour lamp (Kelvin, Bottomley and Baird), which was housed in a metal box fitted with a Wood's Glass window. The solutions were contained in thin-walled Monax Glass tubes of 8 mm. diameter. The exposure required was 2 minutes with a slit width of 0·125 mm. The plates were developed according to the time and temperature table supplied with the developer. Under these conditions a solution of 0·1 μg. of benzpyrene per ml. of petroleum ether gave a reproducible spectrum that was just visible on the photographic plate.

### *Reagents.*

*Benzene, non-fluorescent.*—Pure benzene washed three times with concentrated sulphuric acid, dried and distilled.

*Analar petroleum ether* (b.p. 60°–80°).

*Spence Type H. alumina.*—Activated alumina loses activity when exposed to air. The absorptive power of a particular sample appeared to be correlated with the pH of a suspension in distilled water. For the present purpose, samples showing a pH value of 10·0–10·3 were found to be most suitable.

3:4-*Benzpyrene.*—The commercial product contains a pale-blue-fluorescing impurity which is very strongly adsorbed on alumina. The sample used was purified by absorption on alumina, elution with benzene and recrystallization. Stock solution of benzpyrene: 10 mg. dissolved in 100 ml. benzene. Dilute solution, 1 $\mu$g./ml.: 1 ml. of stock solution diluted to 100 ml. with petroleum ether. Standard solutions were prepared by diluting 8, 6, 4, 2 and 1 ml. respectively to 10 ml. with petroleum ether. The solutions appeared to be quite stable.

The first attempts to separate benzpyrene from soot were based on the method described by Berenblum (1945) for the extraction of benzpyrene from coal tar. In the original method vacuum distillation of the suitable fraction of tar was followed by solution of the distillate in concentrated sulphuric acid. Attempts at vacuum distillation of benzene extracts of soot showed, however, that the composition of the soot extract was very different from that of coal tar. Evaporation of the benzene extract yielded a residue consisting mainly of non-volatile material, which appeared to decompose on distillation *in vacuo* with evolution of gas.

Direct sulphuric acid extraction of the crude tar extracted from soot was also unsuccessful, probably owing to considerable sulphonation. Moreover, recovery experiments, with microgram quantities, of pure benzpyrene from ice-cold sulphuric acid showed that considerable loss of the hydrocarbon occurred, and that the method was useless as a quantitative one for such small amounts.

Chromatographic adsorption was next tried. When an extract of soot was submitted to chromatographic separation, the characteristic fluorescence spectrum of benzpyrene was given by the appropriate eluate. Experiments with various samples of alumina showed that Spence's Type H alumina, containing adsorbed benzpyrene, could be washed freely with a mixture of benzene-petroleum ether (1:10) without elution, and that the benzpyrene was eluted only when the proportion of benzene was increased (3:10). Some fluorescent impurities remained in this eluate, but by suitable dilution with petroleum ether, spectra could be obtained showing benzpyrene bands reasonably free from background (Fig. 4). The benzpyrene content was then estimated by visual comparison with a set of standards (Fig. 1), photographed and developed under the same conditions (Berenblum and Schoental, 1942). Experiments upon photometric comparison are in progress.

The details of a typical experiment are as follows:

1 g. of mixed domestic soot (from a chimney sweep's stock) was extracted in a Soxhlet apparatus. It was found that by refluxing for about 5 hours with 100 ml. of a 1:1 mixture of benzene-petroleum ether, the benzpyrene could be

---

DESCRIPTION OF PLATES.

FIGS. 1–4.—Identification of 3:4-benzpyrene by means of the fluorescence spectrum.
FIG. 1.—3:4-Benzpyrene standards in petroleum ether.
FIG. 2.—Soot eluates of increasing benzene content.
FIG. 3.—Comparison of soot eluates 4 and 5 with benzpyrene.
FIG. 4.—Petroleum ether dilutions of eluate 4 for comparison with standard.

extracted without some part of the other hydrocarbons present. The material extracted amounted to 18 per cent of the soot. The extract was given a preliminary purification by passing it through a short column of alumina (14 × 25 mm.) and eluting with the same mixture of solvents. The solvent was then distilled off, the residue dissolved in 20 ml. of petroleum ether and adsorbed on a column of alumina (14 × 110 mm.). On developing with petroleum ether two purple bands were visible under ultra-violet light. The lower band was easily eluted with benzene-petroleum ether (1:10), while the upper could only be washed down when the benzene content of the eluting fluid was increased (3:10). This latter fraction is shown in Fig. 2, as Eluate 4, total volume 58 ml. Continued elution with the same solvent resulted in an eluate (Fig. 2, Eluate 5), which on dilution (Fig. 3) gave a spectrum no longer identical with that of benzpyrene (Fig. 3, Eluate 5 diluted). The difference is not due to any change of solvent (Chalmers, 1938), as the eluting and diluting fluids for (4) and (5) were the same. Fifty-fold dilution of the 4th eluate (Fig. 4) gave a spectrum in which the intensity of the bands approximated to that of a benzpyrene standard containing 0·1 μg./ml. Since this was the only fraction containing benzpyrene, the benzpyrene content of the original soot appeared, by this method, to be 290 mg./kg., or 0·029 per cent.

A series of analyses upon the same sample of domestic soot are shown in Table I.

TABLE I.

| Mixed domestic soot from chimney sweeps stock. | 3:4-Benzpyrene (%). |
|---|---|
| Preliminary analyses | 0·045 |
| | 0·027 |
| | 0·030 |
| | 0·024 |
| Technique finally adopted | 0·029 |
| | 0·025 |
| Mean | 0·030 |

The results are consistent with one another, and experiments on the absolute quantitative accuracy are in progress.

SUMMARY.

1. 3:4-Benzpyrene has been identified by means of fluorescence spectroscopy as a component of tar obtained by extraction of domestic soot with a benzene-petroleum ether mixture.

2. Adsorption of the extracts on alumina and fractional elution with benzene-petroleum ether yielded solutions suitable for tests of quantitative recovery of benzpyrene by measurement of its fluorescent spectrum.

3. A mixed sample of domestic soot showed a benzpyrene content of about 300 mg./kg. when examined by this method.

Grants from the Medical Research Council and the British Empire Cancer Campaign have supported this work. We should like to thank Dr. I. Hieger for advice on the spectroscopic work, and Professor Sir Ernest Kennaway for his encouragement. Our thanks are also due to Dr. W. R. S. Garton for technical advice on the use of the spectrograph.

### REFERENCES.

Berenblum, I.—(1945) *Nature*, **156**, 601.

*Idem* and Schoental, R.—(1942) *Biochem. J.*, **36**, 86.—(1947) *Brit. J. Cancer*, **1**, 157.

Chalmers, J. G.—(1938) *Biochem. J.*, **32**, 271.

Cohen, J. B., and Ruston, A. G.—(1925) 'Smoke. A Study of Town Air.' London (Edward Arnold & Co.).

Cook, J. W., Hewett, C. L., and Hieger, I.—(1933) *J. chem. Soc.*, 395.

Hieger, I.—(1946) *Cancer Res.*, **6**, 657.

Kennaway, E. L., and Kennaway, N. M.—(1947) *Brit. J. Cancer*, **1**, 260.

Passey, R. D.—(1922) *Brit. med. J.*, ii, 1112.

0.1 μg/ml.
0.2 μg/ml.
0.4 μg/ml.
0.6 μg/ml.
0.8 μg/ml.
1 μg/ml.

FIG. 1.

Eluate 6.

50% benzene.

Eluate 5.

30% benzene.

Eluate 4.

30% benzene.

Eluate 3.

10% benzene.

Eluate 2.

10% benzene.

Eluate 1.

petroleum ether.

FIG. 2.

Hg

Lines

3650

Benzpyrene

Eluate 5. diluted.

Eluate 4. diluted.

Benzpyrene.

Solvent only.